# Deep Learning-Based Classification and Feature Extraction for Predicting Pathogenesis of Foot Ulcers in Patients with Diabetes

**DOI:** 10.3390/diagnostics13121983

**Published:** 2023-06-06

**Authors:** V. Sathya Preiya, V. D. Ambeth Kumar

**Affiliations:** 1 Department of Computer Science and Engineering, Panimalar Engineering College, Anna University, Chennai 600123, India; 2 Department of Computer Engineering, Mizoram University, Aizawl 796004, India

**Keywords:** foot ulcer, deep learning, DRNN, PFCNN, U++net, DM, DFU, risk prediction

## Abstract

The World Health Organization (WHO) has identified that diabetes mellitus (DM) is one of the most prevalent disease worldwide. Individuals with DM have a higher risk of mortality, and it is crucial to prioritize the treatment of foot ulcers, which is a significant complication associated with the disease, as they lead to the development of plantar ulcers, which results in the need to amputate part of the foot or leg. People with diabetes are at risk of experiencing various complications, such as heart disease, eye problems, kidney dysfunction, nerve damage, skin issues, foot ulcers, and dental diseases. Unawareness of the risk associated with diabetic foot ulcers (DFU) is a significant contributing factor to the mortality of diabetic patients. Evolving technological advancements such as deep learning techniques can be used to predict the symptoms of diabetic foot ulcers as early as possible, which helps to provide effective treatment to DM patients. This research introduces a methodology for analyzing images of foot ulcers in diabetic patients, focusing on feature extraction and classification. The dataset used in this study was collected from historical medical records and foot images of patients with diabetes, who commonly experience foot ulcers as a major complication. The dataset was pre-processed and segmented, and features were extracted using a deep recurrent neural network (DRNN). Image and numerical/text data were extracted separately, and the normal and abnormal diabetes ranges were identified. Foot images of patients with abnormal diabetes ranges were separated and classified using a pre-trained fast convolutional neural network (PFCNN) with U++net. The classification procedure involves the analysis of foot ulcers to predict their pathogenesis. To assess the effectiveness of the proposed technique, the study presented simulation results, including a confusion matrix and receiver operating characteristic curve. These results specifically focused on predicting two classes: normal and abnormal diabetes foot ulcerations. The analysis yielded various parameters, including accuracy, precision, recall curve, and area under the curve. The main goal of the study was to introduce an novel technique for assessing the risk of foot ulceration development in patients with diabetes, leveraging the analysis of foot ulcer images. The researchers collected a dataset of foot images and medical data from historical records of patients with diabetes and pre-processed and segmented the data. They then used a deep recurrent neural network to extract features from the segmented data and identified normal and abnormal diabetes ranges based on numerical and text data. Foot images of patients with abnormal diabetes ranges were classified using a pre-trained fast convolutional neural network with U++net to examine foot ulcers and forecast the development of the risk of diabetic foot ulcers (DFU). The study assessed the accuracy of the proposed technique as 99.32% by simulating results for feature extraction and the classification of diabetic foot ulcers. A comparison was made between this proposed technique and existing approaches.

## 1. Introduction

High-throughput computing and developments in the field of biotechnology consistently contribute to affordable data production and a faster way of analysing data using big data technology in biological research. The aim is to build a faster-responding framework that considers the fast-growing biotic data and provides quick an probable responses to basic queries in the medical and biological areas. To achieve efficiency and reliability, choosing the correct approach to identify a pattern and create an efficient model from the dataset given is essential. Research on the prognosis and treatment of diseases that pose a threat to human life is crucial, and diabetes mellitus (DM) is one of the most significant diseases falling under this category. Statistical data from national science of medicine showed that, as of 2019, the number of individuals with diabetes in India was estimated to be 77 million, and is projected to exceed 134 million by 2045, with approximately 57% of cases remaining undiagnosed. Type 2 diabetes, which constitutes the majority of cases, can result in complications affecting multiple organs, which can be broadly categorized into micro-vascular and macro-vascular complications. These complications significantly contribute to premature morbidity and mortality rates among diabetic individuals, leading to a reduced life expectancy, financial burden, and other costs, which impose a profound economic burden on the Indian healthcare system. The cause of diabetesis not fully understood, although researchers suggest that both genetic and environmental factors may contribute to its development [1]. Although DM cannot be cured, medication and drugs can assist in its management. The timely detection and management of diabetes mellitus can help prevent complications and reduce the risk of severe health problems [2]. It can be diagnosed in two ways. The first way is manual diagnosed by a medical practitioner and the second is by using automated instruments. Each way has its own advantages and disadvantages. Manual diagnosis enables healthcare providers to depend on their skills without the need for machine intervention [3]. However, in the initial stages of DM, symptoms may be so mild that even an experienced doctor may not be able to identify them [4].

Dysregulated glycemia can lead to both acute and chronic complications. Diabetes mellitus is associated with an increased predisposition to diabetic heart disease (DHD), a cardiac pathology specific to individuals with diabetes. DHD is associated with several health issues, such as high cholesterol and high blood sugar, significantly increasing the risk of stroke or heart attack [5]. However, people with diabetes may not experience any symptoms due to the nerve damage that affects the nerves controlling the heart. DHD is a leading cause of death among individuals with diabetes, particularly women, who are often underdiagnosed for heart disease. Young or diabetic women are especially vulnerable, and many heart attacks in women are not accurately diagnosed [6]. Studies have found that women with diabetes have a risk of sudden death equivalent to half of that in men suffering from diabetes. Patients with type 2 diabetes are at from two- to four-fold higher risk of developing DHD as compared to those without diabetes [7].

Diabetic foot ulcers are a common issue among many diabetic patients, and if left untreated, they can result in partial or total amputation [8]. Early detection and treatment of these ulcers can prevent their development. However, traditional methods of diagnosis can be inadequate, as diabetic patients often experience a gradual loss of sensation, making self-examination difficult. Furthermore, currently, no automated system exists for the timely identification of diabetic foot ulcers. Prior research has demonstrated an association between elevated temperature and the onset of these ulcers [9].

Various algorithms are employed for the timely detection of diabetic foot ulcers via temperature evaluation. These algorithms encompass infrared images, liquid crystal thermography (LCT), and an infrared (IR) thermometer, and the temperature sensors are incorporated in the weighing scale. To predict DHD at an early stage, various data mining technologies and machine learning techniques are employed [10]. With the advancement of machine learning (ML) and artificial intelligence (AI), an automated program that assists in the early detection and diagnosis of diseases is becoming more viable and efficient than manual methods. The benefits of using automated programs include reducing the workload of medical professionals and minimizing the risk of human error [11]. Computer-based decision support systems can play a crucial rolein facilitating effective diagnosis and management.

The field of DM generates large amounts of data through laboratory evaluations, patient reports, treatments, follow-ups, medications, etc. However, manually organizing all these data can be challenging and lead to poor data quality. To address this issue, hospitals are now utilizing automated devices to perform collation and sharing through the information systems [12]. Automated systems are not only more efficient but also more reliable than manual discovery and analysis. Therefore, it is crucial to develop automated DM systems using machine learning or artificial intelligence techniques. While these methods have their own advantages and limitations, the use of explainable AI systems can improve trust in future AI systems. Causality is also a crucial factor, which can be assessed based on efficacy, usefulness, and transparency for end-users. In recent years, researchers have employed machine learning (ML) and artificial intelligence (AI) techniques to personalize the management and control of diabetes mellitus [13].

The following Section 2 in this paper presents a comprehensive overview of the existing research on DF images. The system model and finer points of the proposed approach are elaborated in Section 3, while Section 4 provides a discussion and results analysis of the model. Finally, a discussion of the conclusions of this study is presented, and potential directions for future research are outlined, in Section 5.

## 2. Related Works

Research studies indicate that skin temperature variations can be measured through the use of thermal images to detect diabetic foot ulcer infection. A number of authors have utilized thermal foot images to distinguish diabetic foot conditions characterized by sympathetic skin reaction from those without a sympathetic skin reaction [14]. Furthermore, the findings of this study demonstrate thermal foot imaging’s efficiency in detecting the presence of diabetic foot diseases, monitoring their progression and evaluating the effectiveness of related treatments [15]. A method of achieving this involves the division of images into six regions of interest (ROIs): arch, heel, forehead, lateral sole, hallux, and lesser toes, followed by the mean calculation of temperature differences for comparison with healthy participants’ temperatures. In a previous study, the authors proposed a system that utilized thermal images to diagnose diabetic foot conditions through the computation of corresponding point temperatures in both feet [16]. The findings indicated that the average temperature among patients with neuropathy ranged between 32.8 and 27.9 °C. Additionally, in another study, the author presented a system for detecting diabetic foot ulcers, which entailed the application of image-processing techniques such as segmentation, registration, and abnormality detection for thermal images. The process of segmenting the foot image involved the utilization of active contours, while background removal was achieved through the application of B-spline non-rigid registration, resulting in alignment [17]. The final step in the process involved the detection of abnormalities by subtracting the level of intensity in the corresponding position from the left foot to the right foot. Specifically, if the temperature difference exceeds a predefined threshold, it is deemed indicative of an abnormal region, as per the findings of this study.In another study, infrared images were employed for the discrimination of diabetic foot ulcer classes: those with no visible signs, those with local complications, and those with diffuse complications [18]. The mean temperature difference between the ipsilateral and contralateral foot can be calculated to accomplish this. The researcher utilized thermal imaging to detect abnormalities in diabetic foot conditions by correlating area temperatures with color codes. The approach entailed employing a rainbow palette, which was segmented into 10 distinct colors based on differences between them [19]. MATLAB mobile platform and Android smartphone were employed in the study to create an Android-based thermal system. Based on a comparison with a normal test image, the system predicted ulcers from four different ROI areas. Despite this, the study did not focus on cases that carried a high rate of complications, relying only on static thermal information in predicting ulcers in diabetics with grade-zero foot conditions [20]. The use of infrared images for detecting diabetic foot conditions through the application of image processing techniques was explored in a previous study [21], which reported the highest accuracy rate of 95.66%. However, the latitude of this research was limited to detect abnormalities. In the study conducted by [22], they utilized an alternate convolutional neural network to classify diabetic foot ulcers. Meanwhile, in [23], a thermal system was proposed to detect abnormalities in diabetic foot by extracting the textural and entropy features from the decomposed discrete wavelet transform (DWT) and higher-order spectra (HOS). However, the study was limited in that it could not distinguish between different types of diabetic foot conditions, achieving an identification rate of 89.39%. In [24], the authors focused on developing an ensemble model that utilized a majority voting technique to combine unweighted predictions from various machine learning models. On the other hand, the authors of [25] focused on discriminating techniques, where they combined the basic classifiers through a process that could adjust to the input observations and output requirements of each individual learning system.In the training set, the type of combination employed could be optimized by assigning weight to each classifier in order to improve the combined performance. In a study by the authors of [26], ensemble-based methods were recommended as the most effective approach for data stream classification problems. Furthermore, the authors compared ensemble learning with sixty other algorithms. Similarly, the author of [27] notes that in the realm of machine learning, multiple classifier systems have undergone considerable development in recent years, presenting promising solutions to a range of problems and enhancing precision.

## 3. Proposed Work

### 3.1. Architectural Framework

**Figure 1:**
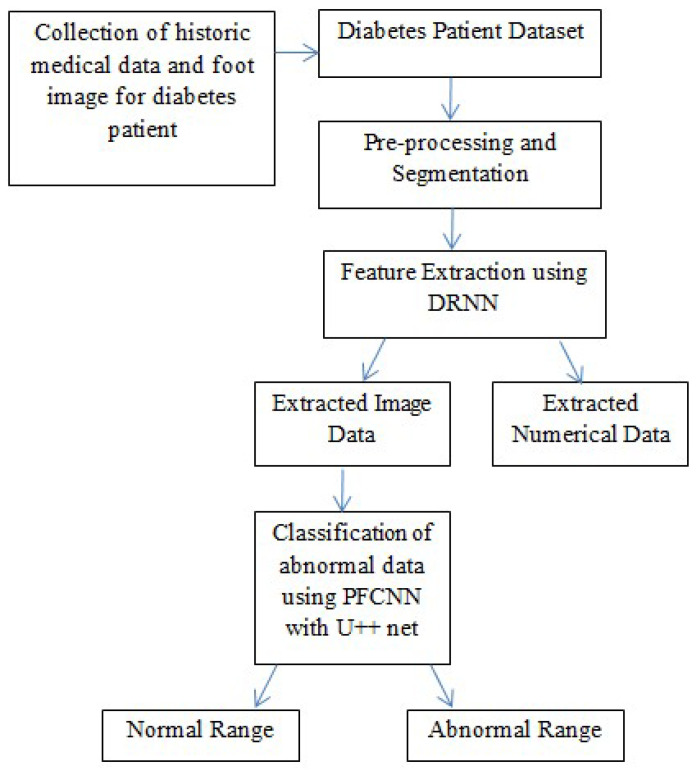
Flow Schematic Diagram for Foot Ulcer Classification.

The technique presented in this research endeavors to identify the risk of diabetic foot ulcers in patients with diabetes through the analysis of foot ulcer images, as illustrated in Figure 1. In this study, the DFU2020 dataset was utilized to extract the feature set from foot ulcer images and classify them as either normal or abnormal for patients with diabetes. The dataset consisted of both numerical and image data, which were pre-processed and segmented, and feature vectors were extracted using advanced deep neural networks, including deep recurrent neural networks (DRNN) for numerical data and deep neural networks (DNN) for image data. To detect the level of diabetes, the numerical feature vectors were analyzed, and if the diabetes range was higher, appropriate treatment was provided. The diabetic foot images of the patients were subsequently employed for the classification task, aiming to predict the pathogenesis of foot ulcers. However, if the diabetes range was normal, the data were updated in the database for future reference to identify nerve damage and predict the development or progression of foot ulcers. The foot ulcer images of patients with abnormal diabetes ranges were classified using a pre-trained fast convolutional neural network (PFCNN) with U++net. The classification results helped to predict the risk of foot ulcers. Figure 1 illustrates the flow schematic diagram for foot ulcer classification and provides a comprehensive overview of the entire process.

### 3.2. Feature Extraction Using Deep Recurrent Neural Network (DRNN)

**Figure 2:**
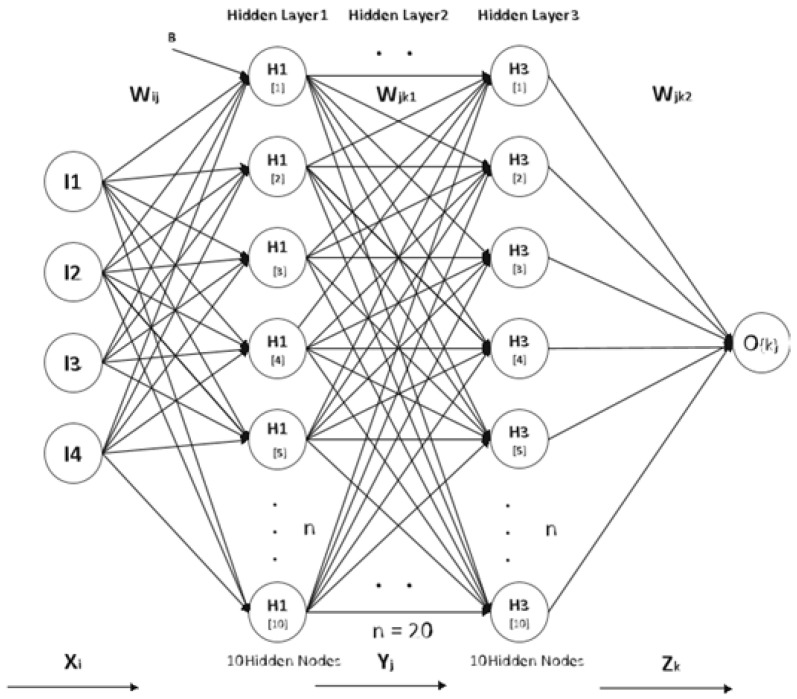
DRNN architecture.

Utilizing the depiction of deep neural networks (DNN), we formulated the computational protocols for the forward and backward propagation, which serve as quintessential constituents of both the inference and training phases. A schematic of the DRNN architecture can be found in Figure 2.

As a general rule, an a-by-b block matrix and the weight matrix are assumed to be $W\in R_{a\times b}$ for a fully connected layer in a deep neural network (DNN) block matrix. This matrix, *W*, comprises several diagonal sub-matrices of size p×p. Since a total of $\frac{m}{p}\times \frac{n}{p}$ sub-matrices of size $\frac{m}{p}\times \frac{n}{p}$ exist, every sub-matrix involved in the investigation possesses its individual permutation parameters, and each of them is assigned an index based on their position, which varies from 0 to the product of $\frac{m}{p}$ and $\frac{n}{p}$ subtracted by 1. The permutated parameter value for the sub-matrix located on the *l*th diagonal is denoted as $k_{l}$. Consequently, any value of $w_{ij}$ in *W* can be represented in the following manner:

(1) $$w_{ij}=\left\{\begin{array}{c} qk_{l\times p+c}\;\;\;\mathrm{if}\;\left(c+k_{l}\right)\;\mathrm{mod}\;p\equiv d\;\\ \mathrm{otherwise} \end{array}\right.$$

The variables *c* and *d* are congruent to *i* and *j* modulo *e*, respectively. The value of *l* can be computed as the product of (i/p) and (n/p)+(j/e). The vector *m*, which includes all non-zero elements of *W*, is represented as $q=(q_{0},q_{1},\ldots ,q_{mn/p-1})$. Feature extraction using a DRNN is illustrated in Figure 2.

In the FC layer’s inference phase, $y=\psi \left(a\right)=\psi {\left(Wx\right)}^{4}$ is used for forward propagation. In this context, the activation function is represented by ψ(·), and $a=Wx={\left(a_{1},a_{2},\ldots ,a_{m}\right)}^{T}$, where $x={\left(x_{1},x_{2},\ldots ,x_{n}\right)}^{T}$ denotes the input vector, and $y={\left(y_{1},y_{2},\ldots ,y_{m}\right)}^{T}$ represents the output vector. The activation function plays a crucial role in this scenario. To streamline the calculation of $a_{i}$ using the block diagonal weight matrix *X*, which is trained and comprises entries as per Equation (2), equation $\sum_{g=0}^{n/p-1}w_{ij}x_{j}$ is applied. The values of *j* are determined by the formula j≡(i+kl)modp+gp, with l=g+(i/p)×(n/p). During the inference phase, weight storage necessitates only the mn/p minus the length of the vector *q* that includes all the non-zero entries of *X*. This significantly simplifies the original computation, as represented by Equation (2).

(2) $$a_{i}=\sum_{g=0}^{n-1}w_{ij}x_{j}$$

In order to retain the diagonal structure of the fully connected layer in a deep neural network throughout each iteration of the training phase, it is essential to include backward propagation. During forward propagation, the value of *y* is ψ(a) and the *a* take the values of ${\left(a_{1},a_{2},\ldots ,a_{m}\right)}^{T}$. During the backward propagation process used to compute the gradient, the equation of $\frac{\partial J}{\partial w_{ij}}=x_{j}\frac{\partial J}{\partial a_{i}}$ is utilized, where *J* denotes the loss function of the neural network. As per the backward propagation principle, the present layer’s $t^{th}$ is transmitted to the prior layer as $a^{\left(t-1\right)}$. Consequently, using the above equation, the updating rule for weight to the fully connected layer of DRNN can be derived.

(3) $$w_{ij}\leftarrow w_{ij}-\in x_{j}\frac{\partial J}{\partial a_{i}},\;\mathrm{for}\;\mathrm{any}\;w_{ij}\neq 0$$

(4) $$\frac{\partial J}{\partial x_{j}}=\sum_{g=0}^{m/p-1}w_{ij}\frac{\partial J}{\partial a_{i}}$$

The calculation of *i* is determined by the congruence of (j+p−kl)modp and adding it with gp; here, the *l* is calculated as the sum of the ratio of gn to *p* and the ratio of *j* to *p*. The rate of learning is represented as η. In Equation (3), by utilizing Equation (4), it is feasible to streamline the computation of $\frac{\partial J}{\partial a_{i}}$ via the backward propagation of the current layer’s $x_{i}$ as $a_{i}$ to the preceding layer. It is worth highlighting that Equations (3) and (4) guarantee that the weight update procedure always culminates in a sparse network that exhibits a block diagonal structure. This, in turn, yields a practical and efficient end-to-end training solution.

### 3.3. Inference of CONV Layer in Forward Propagation

In convolutional neural networks (CNNs), the weight tensor of a CONV layer can be viewed as a “macro”-matrix where each entry represents a filter kernel. Inspired by the diagonal structure of weight matrices in fully connected (FC) layers, a similar approach can be applied to the 4D weight tensor of a CONV layer. This can introduce diagonal dimensions to both the inward and outward channels of the weight tensor. Using this approach, a diagonal matrix can be used to specify the forward propagation of the CONV layer, similar to the method used in the FC layer. The fundamental idea behind this approach is that a diagonal matrix can simplify the computation of the output layer, and this methodology is applicable to both FC and CONV layers.

(5) $$Y\left(i,x,y\right)=\sum_{a=0}^{c2/p-1}\sum_{w=0}^{w_{1}-1}\sum_{h=0}^{h_{1}-0}F\left(i,j,w,h\right)ℵ\left(j,x-w,y-h\right)$$

According to Equation (5), the convolutional layer involves an input tensor *X* belonging to $R_{c0\times w0\times h0}$, an output tensor *Y* belonging to $R_{c2\times w2\times h2}$, and a weight tensor *F* belonging to $R_{c0\times c2\times w1\times h1}$. Here, the width and height of the input, kernel, and output tensors are denoted by $w_{i}$ and $h_{i}$, where *i* takes the value of 0, 1, and 2, respectively. Furthermore, c0 indicates the number of input channels and c2 indicates the number of output channels. It is also worth noting that *l* is calculated as *g* added to the product of (i/p) and (n/p), where *j* is the congruence of (i+kl) mod *p* + gp.

### 3.4. Training the CONV Layer in Backward Propagation

For the preservation of the diagonal structure of the convolutional layer in DNN during the training phase, it is necessary to modify the weight updating process. This can be achieved by following a similar approach to the fully connected layer. Consequently, the rule for updating the weight to propagate backward in the convolutional layer can be expressed as follows.

(6) $$F\left(i,j,w,h\right)\leftarrow F\left(i,j,w,h\right)-\in \sum_{x=0}^{w_{2}}\sum_{y=0}^{h_{2}}ℵ\left(i,x-w,y-h\right)\frac{\partial J}{\partial X(i,x,y)},\;\mathrm{for}\;\mathrm{any}\;F\left(i,j,w,h\right)\neq 0$$

(7) $$\frac{\partial J}{\partial X(i,j,x)}=\sum_{g=0}^{(c_{o}/\rho )-1}\sum_{w=0}^{w_{1}-1}\sum_{h=0}^{h_{1}-1}F\left(i,j,w,h\right)\frac{\partial J}{\partial Y(i,x+w,y+h)}$$

In order to ensure that the diagonal structure of the convolutional layer is maintained while performing the training, the process of updating the weight needs to be re-designed. Equation (6) calculates $\frac{\partial J}{\partial X(i,x,y)}$ and the value of *i* is calculated as (j+p−kl)modp+gp and the value of *l* is calculated as $\frac{gn}{p}+\frac{j}{p}$. It should be noted that this calculation is supported by the calculation in Equation (6). According to Equation (7), every X(j,x,y) present in the current layer will be retroactively propagated to the prior layer as M(j,x,y). The computation in Equation (6) is helpful in this calculation, since each current layer’s X(j,x,y) is propagated backward into the prior layer as M(j,x,y). By updating the weights according to Equations (6) and (7), the sparsely trained network will maintain the block diagonal structure.

**Figure 3:**
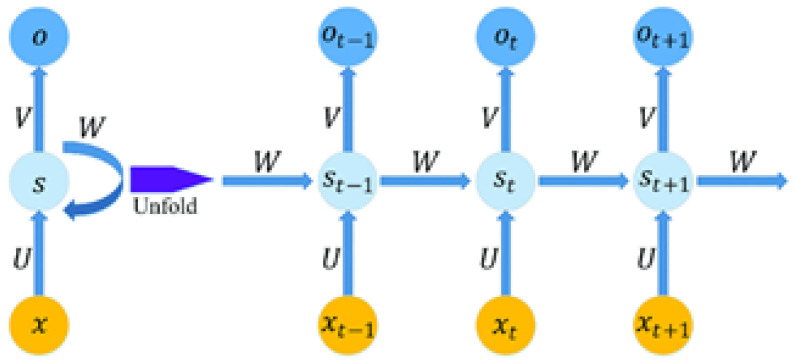
RNN architecture.

After the feature extraction from the image, the numerical/text data of the diabetes dataset are obtained using recurrent neural networks (RNNs). RNNs are similar to fully connected neural networks but with some of their layers refactored into a loop, as illustrated in Figure 3. There are two inputs to this loop, which is iteratively added or concatenated, and a matrix multiplication and non-linear function are applied.

The proposed neural network architecture for processing phrases involves three input layers. The first layer receives a feature vector that contains information about the words in the phrase. This vector can vary in size and is fed into an LSTM layer. The second layer captures contextual information and has a variable length. To capture the context, a window size of n is considered, where n words to the left and right of the phrase are concatenated and fed into the LSTM layer. The last layer is a fixed-length input layer that processes a vector containing general information about the phrase and its context. This vector includes the minimum and maximum coordinate-wise values of a word vector and binary features indicating the presence or absence of various attributes in the phrase. Using a fixed-length sparse vector is not feasible since the length and context of phrases vary. Thus, the proposed architecture employs recurrent neural networks for processing vectors of arbitrary length, ensuring optimal performance.

### 3.5. Pre-Trained Fast Convolution Neural Network (PFCNN) for Classification

**Figure 4:**
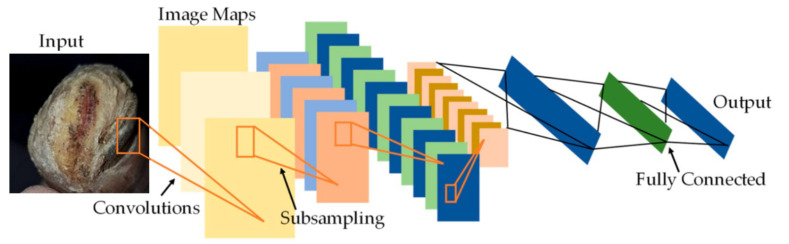
Architecture of PFCNN.

The diabetic foot ulcer is classified into five stages, wherein stage 1 represents a normal foot, emphasizing the importance of maintaining healthy feet and using appropriate footwear while also stressing the need for regular foot care. Stage 2 indicates a high-risk foot, where foot ulcers require attention due to the possibility of their developing complications such as neuropathy, ischaemia, deformity, swelling, and callus. Stages 3, 4, and 5 represent ulcerated, infected, and necrotic feet, respectively. Starting from stage 3, the foot is considered ulcerated and requires immediate attention. Stage 3 represents damage to the peripheral nerve, increasing the likelihood of developing neuropathy-related risks, which may eventually lead to cardiovascular disease. Thus, stages 1 and 2 are considered normal, while stages 3–5 are classified as abnormal. For patients with foot images showing an abnormal diabetes range, a PFCNN is utilized as a linear classifier to predict the risk of foot ulcers. During training, the CNNs in the architecture the PFCNN architecture employs the ReLU activation and completes the dropout after each convolutional layer, as the PFCNN model itself uses weighted softmax cross entropy loss with an Adam optimizer. The seven frames of size 60 × 40, centered on the current frame, are used as inputs, with hardwired kernels applied to produce the multiple channels of information. The second layer generates 33 feature maps across five channels: optflow-x, gradient-x, gray, gradient-y and optflow-y. The convolutional layers in CNNs are succeeded by an output layer and one or more fully connected layers. Figure 4 depicts the architecture of the PFCNN. A label weight is calculated by inverting the occurrence of each label in the training set.

It is depicted that the input that goes into a layer *s* of a neural network $I^{\left(m\right)}$. For a pre-trained convolutional layer *m*, the input is a 3D object with dimensions $n_{1}^{\left(m-1\right)}\times n_{2}^{\left(m-1\right)}\times n_{3}^{\left(m-1\right)}$ and $n_{c}^{\left(m-1\right)}$ channels, where $I^{\left(m-1\right)}\in R^{n_{1}^{\left(m-1\right)}\times n_{2}^{\left(m-1\right)}\times n_{3}^{\left(m-1\right)}}$. The elements of the input are represented by $I_{i,j,k}^{\left(m,l\right)}$, where *i*, *j*, and *k* are the index of the 3D volume and *l* picks the channel.

Let the volume of filters or channels produced by a convolutional layer *m*, denoted by $n_{c}^{\left(s\right)}$, along with its dimensions, i.e., $n_{1}^{\left(s\right)}\times n_{2}^{\left(s\right)}\times n_{3}^{\left(s\right)}$, define its output. The result of *m*th layer is produced by convolving its input with a specific filter. This process can be expressed as $I^{\left(m,l\right)}i,j,k=f\tanh\left(b^{\left(m,l\right)}\right)+\sum_{i,j,k,l}I^{\left(m-1,l\right)}i-i,j-j,k-kW^{\left(m-l\right)}i,j,k,l$. In this equation, the parameters $W^{\left(m,l\right)}$ and $b^{\left(m,l\right)}$ define the filter used for the *l*th feature map in layer *m*. The positions where the filters are assessed, which are represented by the variables *i*, *j*, and *k* in the equation $I^{\left(m,l\right)}i,j,k$, as well as the dimensions of the filters, which are represented by the non-zero values of $W^{\left(m,l\right)}$, are both determined by the network architecture parameters. Additionally, the activation function used is a hyperbolic tangent with ftanh(a)=tanh(a).

By preserving the spatial arrangement of the convolutional layer’s input, it gradually constructs increasingly intricate representations of the input as more layers are added. The resulting convolutional layer’s output is subsequently fed into a fully connected network layer. Convolutional layers produce a unified vector output regardless of the spatial and channel structure. Here, the output of a fully connected layer is a 1D vector of $I^{\left(m\right)}$, where the size of the vector is determined by the network architecture parameters. Then, the result of the *i*th neuron in layer *m* is represented using Equation (8).

(8) $$I_{i}^{\left(m\right)}=f_{ReLU}\left(b^{\left(m,i\right)}+\sum_{j}I_{j}^{\left(m-1\right)}W_{j}^{\left(m,i\right)}\right)$$

Within this particular context, the neural network’s $i^{th}$ neuron within $m^{th}$ layer is depicted as the parameters $W^{\left(m,i\right)}$ and $b^{\left(m,i\right)}$. Meanwhile, the summation over *j* encompasses all dimensions of the input. The rectified linear unit (ReLU) is selected as the activation function and represented as $f_{ReLU}\left(\cdot \right)$. It is worth noting that $f_{ReLU}\left(a\right)$ is calculated as the maximum of 0 and *a*.

ReLU is the rectified linear unit activation function, which is popularly utilized across various fields, and its effectiveness is especially noticeable when performing the classification operation because of the sparsity it introduces in the outputs. This sparsity, in turn, assists in creating a distinct boundary between the classes at the learning process. The ultimate input that is received by the output layer is acquired from the concluding fully connected layer, and the design and configuration of the output layer are subject to change based on the specific task that is being executed. In this context, two distinct types of output functions are considered. In problems where classification into *K* classes is required, the softmax function is commonly used as the output function. This can be represented using Equations (9) and (10).

(9) $$f_{i}=\frac{\exp(I_{i}^{\left(o\right)})}{\sum_{j}\exp\left(I_{j}^{\left(o\right)}\right)}$$

(10) $$I_{i}^{\left(o\right)}=b^{\left(o,i\right)}+\sum_{k=1}^{k}W_{k}^{\left(o,i\right)}I_{k}^{\left(N\right)}$$

In this scenario, the index *N* designates the final fully connected layer. The parameters of $i^{th}$ output layer are represented by $b^{\left(o,i\right)}$ and $W^{\left(o,i\right)}$. Taking the inputs into account, the output of class *i*, denoted as $f_{i}$, has a range of [0,1] and is considered to be the probability of the corresponding class. Moreover, an alternative to the logistic output function is also taken into consideration, as shown by Equation (11).

(11) $$f=a+\left(b-a\right){\left[1+\exp\left(b^{\left(o\right)}+\sum_{j}W_{j}^{\left(o\right)}I_{j}^{\left(N\right)}\right)\right]}^{-1}$$

Within this particular context, the continuous output function is represented by *f* and restricted between the values (a,b) through the use of the output function of scaled logistic function. Both parameters $b^{\left(o\right)}$ and $W^{\left(o\right)}$ play a major role in restricting the output range of the function. This logistic output function is commonly known as a scaled logistic output function.

It is worth mentioning that, in the event of a multi-class classification problem based on ranking, such as when predicting the distortion levels, the use of this output function is anticipated to yield superior results.

The following Table 1 details the stride size that was applied to the input volume of the image, from left to right. Once it reaches the right end, the stride is applied again from the leftmost corner to the right of the specified stride pixel, moving down the image from the current position until it has been applied to the entire volume of the image. As shown in the table, the rectified linear unit (ReLU) activation function was utilized to fit the results and achieve higher accuracy. In this case, the input was classified into two classes, which constitute the output from the dense layer.

## 4. Training

The primary goal of this research is to fine-tune the parameters of a pre-existing network architecture to best match the provided data. This is accomplished by specifying an objective function and using gradient-based optimization to determine the network parameters that minimize the objective function. The collection of training examples *D* may be augmented and comprises input *n* and output *y*, represented by $D={n_{i},y_{i}}_{i=1}^{D}$, for each *i*. The aforementioned system accepts input *n*, which has the potential to be a segmented CT scan, and generates an output *y*. The output *y* can function either as a classifier of the input as benign or malignant or as an indicator of the malignancy level. The biases *b* and weights *W* applied to each layer of the network are symbolized by Θ. Then, the objective function will take the form defined using Equation (12).

(12) $$E\left(\Theta \right)=\sum_{i=1}^{D}L\left(y_{i},f\left(n_{i},\Theta \right)\right)+\lambda E_{\mathrm{prior}}\left(\Theta \right)$$

In this context, the function $f(n_{i},\Theta )$ is utilized to compute the output of the network using input $n_{i}$ and the set of parameters Θ. $L(y_{i},f\left(n_{i},\Theta \right))$ is the loss function, which is applied to assess the deviation among the expected output of the network *y* and the predicted output $\hat{y}$. Moreover, the function $E_{\mathrm{prior}}\left(\Theta \right)$, calculated as ${\left|W\right|}^{2}$, signifies the previous decay of weight, which aims to avoid overfitting by imposing a cost on the weight norm. The magnitude of λ determines the intensity of the prior regularization.

This study employs different objective functions based on the selected output function. When using the softmax output function, the function of the standard cross-entropy loss of $L\left(y_{i},\hat{y}\right)=-\sum_{k=1}^{k}y_{k}\log\left(\hat{y}k\right)$ is applied; here, *y* represents a binary indicator vector, and $\hat{y}$ represents a probability vector for each of the *K* classes. However, this loss function is limited by its inability to differentiate between different classification errors. To address this, the function of squared error loss is preferred to the function of scaled logistic function, where $L\left(y_{i},\hat{y}\right)={\left|\left(y_{i},\hat{y}\right)\right|}^{2}$, and both the *y* value and $\hat{y}$ value are the real values. To update the parameters at iteration *t*, equation $\Theta t+1=\Theta_{t}+\Delta \Theta_{t+1}$, is utilized, with $\Delta \Theta_{t+1}=\rho \Delta \Theta_{t}-\epsilon \nabla E_{t}\left(\Theta_{t}\right)$.

The equation shown here outlines the process of updating the network’s parameters while training. The momentum parameter ρ is set to a constant value of 0.9, and the momentum vector $\Delta \Theta_{t+1}$ is calculated using the previous momentum vector $\Delta \Theta_{t}$, the learning rate ϵ, and the gradient of the objective function $\nabla E_{t}\left(\Theta_{t}\right)$ is the computation of this metric, which is predicated based on the selection of training examples during the iteration *t*. In the first iteration, the biases are set to 0, and the filter and weight values are initialized by uniformly sampling from the interval of $\left(-\sqrt[{n}]{\frac{\cdot 6}{f_{an_{in}}+f_{an_{out}}}},\sqrt[{n}]{\frac{\cdot 6}{f_{an_{in}}+f_{an_{out}}}}\right)$; here, the value of $f_{an_{in}}$ corresponds to the number of nodes in the prior hidden layer, while the value of $f_{an_{out}}$ corresponds to the number of nodes in the present layer. The training process entails 2000 epochs, wherein we initialize $\epsilon_{t}$ to 0.01, and decrease the learning rate by 10% after every 25 epochs to guarantee convergence.

## 5. Performance Analysis

**Figure 5:**
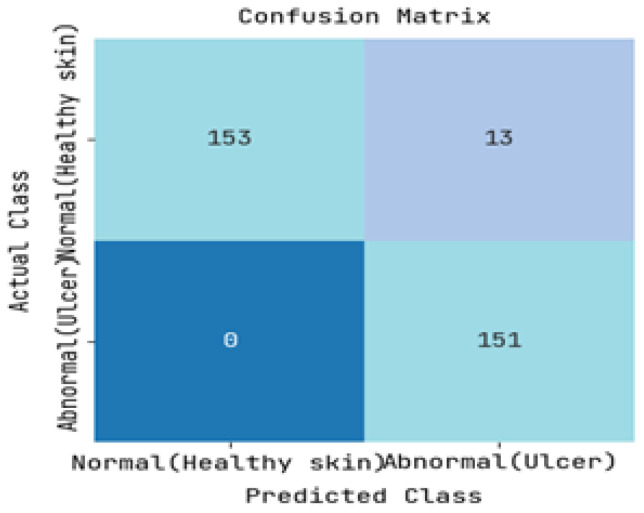
Resulting Confusion matrix of Testing.

**Figure 6:**
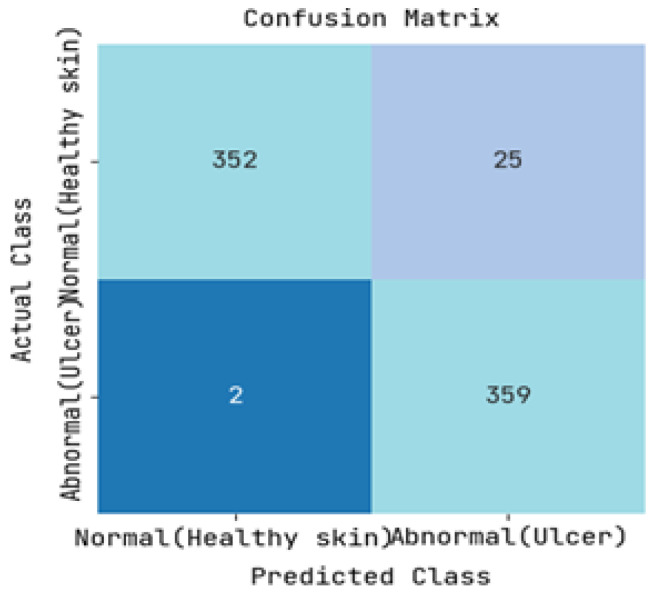
Resulting Confusion matrix of Training.

The DFUC2021 dataset was utilized to study the pathology of diabetic foot ulcers, with a specific focus on infection and ischemia. The dataset comprises a total of 15,683 DFU images, out of which 5955 images were used for training, 5734 images were used for testing, and the remaining 3994 images were left unlabelled. The study’s primary objective was to assess the performance of an image-based risk prediction model of diabetic foot ulcers. The results of this performance analysis are showcased in this section, with Figure 5 and Figure 6 presenting the confusion matrices for diabetic range detection in the test data and train data, respectively. These matrices provide a clear overview of the model’s accuracy in predicting the diabetic range, highlighting the number of true positives, false positives, true negatives, and false negatives.

The use of the DFUC2021 dataset in this research allowed for a comprehensive assessment of the performance of the proposed model, which could aid in developing better diagnostic tools for patients with diabetes. The availability of a large dataset such as DFUC2021 can help researchers better understand the complexities of diabetic foot ulcers and ultimately lead to more effective treatment strategies for patients suffering from this condition.

**Figure 7:**
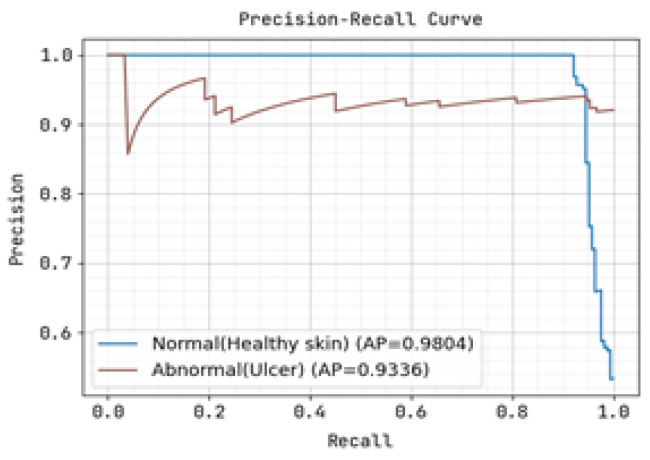
Precision-Recall Curve for Training.

**Figure 8:**
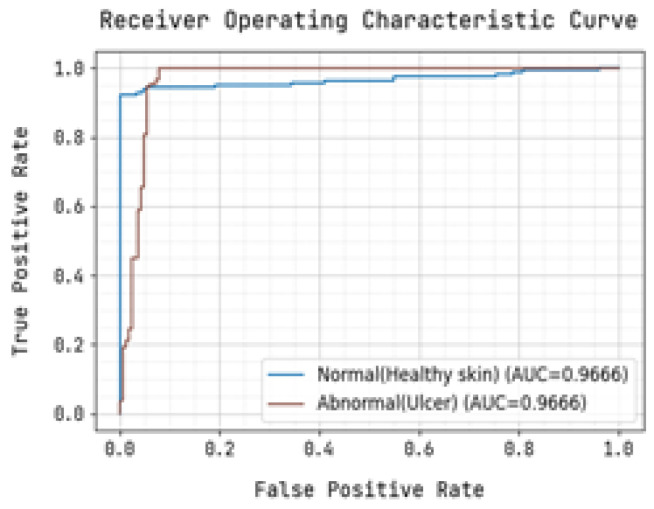
Receiver Operating Characteristic Curve for Training.

**Figure 9:**
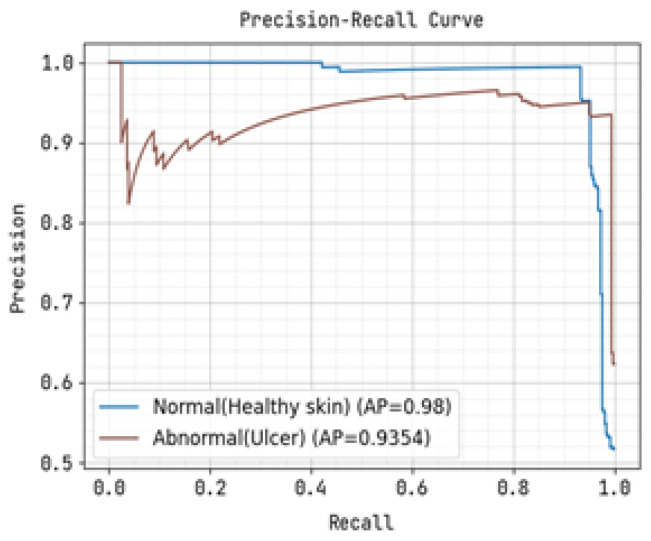
Precision-Recall Curve for Training.

**Figure 10:**
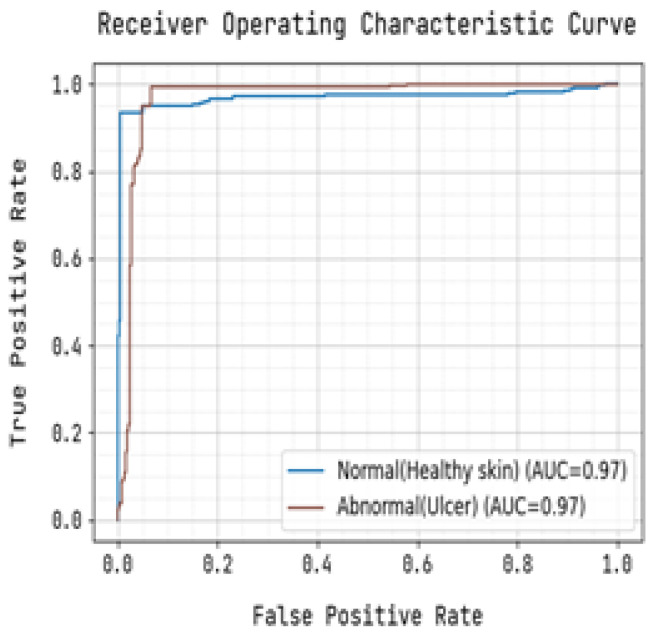
Receiver Operating Characteristic Curve for Training.

The ROC curve and precision-recall curve were generated to distinguish between normal and abnormal foot ulcers through the feature extraction and classification process based on the testing data of foot ulcers, as shown in Figure 7 and Figure 8, respectively. Similarly, Figure 9 and Figure 10 depict the ROC curve and precision-recall curve for distinguishing between normal and abnormal foot ulcers through the feature extraction and classification process based on the training data of foot ulcers.

The determination of the precision-recall curve and the receiver operating characteristic (ROC) curve for classifying the normal and abnormal foot ulcer based on foot ulcer testing data is presented in Figure 7 and Figure 8.

The precision-recall curve and the receiver operating characteristic (ROC) curve used to classify diabetic foot ulcers using foot ulcer training data are determined in Figure 9 and Figure 10, respectively. Table 2 displays the input foot ulcer images used for training, and presents the pre-processing and segmentation steps, followed by the resulting classified output.

To detect anomalous foot ulcers, a pre-trained fast convolutional neural network (PFCNN) incorporating U++net architecture was employed. Consequently, the prediction of foot ulcer pathogenesis becomes simplified. The proposed system achieves a classification performance with precision and recall rates of 0.9354, as revealed by the results.

Table 3 presents a detailed comparison of the proposed classification and feature extraction techniques and the existing methods. When compared with the other models, the proposed method of PFCNN and DRNN attained an accuracy of 99.32.

## 6. Conclusions

This research introduces a novel methodology to automatically identify the pathogenesis of diabetic foot ulcers through image feature extraction and classification techniques. In this study, a dataset of foot ulcer images was employed to extract features using a DRNN-based feature extraction approach. This enabled the differentiation between normal and abnormal diabetes ranges. For classifying foot ulcer images within the normal and abnormal diabetes range, a PFCNN-based classification method was utilized. The classification accuracy served as an indicator to identify the risk of foot ulcer pathogenesis. The proposed architecture presents a valuable tool for detecting abnormal diabetes ranges and assessing the risk of foot ulcers. Notably, the performance of the proposed technique demonstrates an improved classification accuracy, surpassing previously recorded values. This improvement can be attributed to the effective isolation technique that was employed and the increased number of statistical features. The objective of this study is to develop an automated approach for diagnosing the risk of diabetic foot ulcers using foot ulcer classification and feature extraction techniques. The proposed method utilizes DRNN-based feature extraction and PFCNN-based classification to distinguish between normal and abnormal diabetes ranges and predict the severity of foot ulcers. The results showcase a significant enhancement in classification accuracy, reaching 99.32%, when compared to previous studies. This improvement can be attributed to the augmented number of statistical features and the utilization of an effective isolation technique. 

## Figures and Tables

**Table 1:** Architecture of the CNN model.

Layer	Params	Activation	Output
Input			28×28×28
Conv 1	5×5×5	ReLu	28×28×28×7
Max Pool	1×1×1, stride 2×2×4		14×14×7×7
Conv 2	5×5×3	ReLu	14×14×7×17
Max Pool	2×2×2, stride 1×1×0		6×6×3×17
Dense			256
Dense			2

**Table 2:** Output of Classification.

S.NO	Input Image	Pre-Processing	Segmentation	Classified Output
Abnormal Ulcer	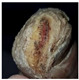	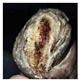	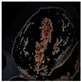	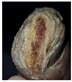
	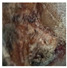	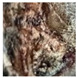	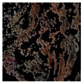	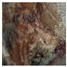
	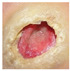	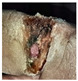	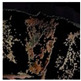	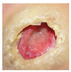
	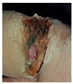	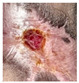	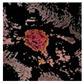	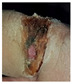
Normal (Healthy skin)	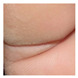	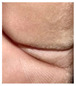	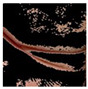	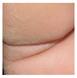
	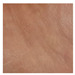	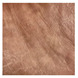	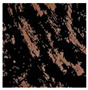	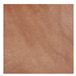
	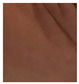	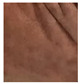	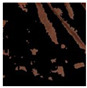	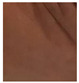
	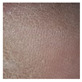	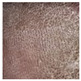	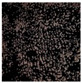	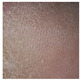

**Table 3:** Comparison of Classification and Feature Extraction with Proposed Method and Existing Methods.

Author	Classification Technique	Feature Extraction Method	Accuracy
Xu Y et al. [5]	Class Knowledge Bank(CKB)	DNN-MLP	79%
Haque et al. [6]	K-Means Clustering	Chi-square, mrmr, Relief Algorithm, FSCNCA	96%
Ahsan et al. [3]	End to end CNN	DFUNet	98.49%
Thotad et al. [2]	EfficientNet	DFUNet	98.97%
Proposed work	Pre-trained Fast Convolution neural network (PFCNN)	Deep recurrent neural network (DRNN)	99.32%

## Data Availability

The foot ulcer images obtained from Kaggle were utilized for the purpose of this research.
